# The combined effect of dietary live microbe intake and physical activity on overweight or obesity in children and adolescents aged 2–17 years: a cross-sectional study from the NHANES

**DOI:** 10.3389/fped.2025.1653786

**Published:** 2025-12-01

**Authors:** Shanhu Wang, Xiaoli Chen, Yangyang Wu

**Affiliations:** Department of Pediatrics, The Second Affiliated Hospital of Wenzhou Medical University, Wen Zhou, Zhe Jiang, China

**Keywords:** dietary live microbe intake, physical activity, overweight, obesity, children

## Abstract

**Aim:**

The study aimed to explore the combined effect of dietary live microbe intake and physical activity on overweight or obesity in children and adolescents aged 2–17.

**Methods:**

Data of children and adolescents aged 2–17 were obtained from the National Health and Nutrition Examination Surveys (NHANES) database in 1999–2020. Dietary live microbe intake was assessed through 24-h dietary recalls and categorized into three levels: low, medium, and high (with “MedHi” referring to the combined medium and high intake groups for analysis). Physical activity level was determined by self-reported using the questionnaire. Overweight and obesity was evaluated using the body mass index-for-age percentile growth charts. Weighted univariate and multivariate logistic models were conducted to explore the associations between dietary live microbe intake, physical activity, and overweight or obesity in children and adolescents. With odds ratios (ORs) and 95% confidence intervals (CIs) presented.

**Results:**

Among the included children and adolescents, 10,086 had overweight or obesity. We found that those with high live microbe intake (from the MedHi group) and ideal physical activity had the lowest incidence of overweight or obesity (*χ*^2^ = 52.311, *P* < 0.001). Specifically, children and adolescent with high live microbe intake and ideal physical activity were related to a lower occurrence of overweight or obesity (OR = 0.65, 95%CI: 0.54–0.77). The expression levels of enterodiol and enterolactone were the highest in the high live microbe intake and ideal physical activity group. CRP expression levels were lowest in high live microbe intake and ideal PA group.

**Conclusion:**

Dietary live microbe intake and physical activity has a potential combined effect on overweight or obesity in children and adolescents. Further longitudinal studies are needed to confirm the causal relationships and explore the mechanisms.

## Introduction

Childhood obesity has emerged as a major global public health concern, with recent estimates indicating that over 206 million children and adolescents aged 5–19 years are overweight or obese globally—a trend that has been accelerating in both developed and developing countries (1). Childhood obesity could persist into adulthood and is linked to a variety of detrimental health outcomes, including cardiovascular disease, complications, and mental health disorders (2, 3). Understanding the contributing factors to childhood obesity has become paramount for designing effective prevention and intervention strategies. Among the various factors implicated, dietary habits and physical activity (PA) levels have long been identified as key determinants of body weight regulation.

Dietary patterns, especially those rich in high-calorie, low-nutrient foods, are central to the rise in childhood obesity. However, growing evidence suggests that the gut microbiota, influenced by the consumption of dietary live microbes such as probiotics, may play an important role in metabolic health and weight regulation (4, 5). Live microbes are microorganisms that, when ingested in adequate amounts, confer health benefits to the host (6). Foodborne microbes’ composition influences the gut microbiota (7). In 2022, Marco et al. (8) quantified live microbes (per gram) for 9388 food codes contained in 48 subgroups in the National Health and Nutrition Examination Surveys (NHANES) database and categorized dietary live microbe intake into low, medium, and high levels. For this study, we focused on the combined moderate and high intake groups, termed “MedHi”. Negative links have been found between dietary live microbe intake and obesity or cardiovascular disease (9, 10). Yet, the impact of live microbes on childhood obesity remains inconclusive, and most existing evidence has examined live microbe intake in isolation, particularly regarding the interaction between live microbe intake and other lifestyle factors such as PA. This separate approach may overlook the potential for combined effects.

PA has long been acknowledged as a crucial factor in weight management due to its positive effects on energy expenditure, muscle mass, and metabolic function (11). Similarly, PA significantly influences the gut microbiota (12, 13). Supplementation with food-derived microbial communities, combined with active PA, may help alleviate systemic inflammation (14). However, the interaction between PA and the gut microbiome remains an underexplored area in pediatric research, and crucially, the combined role of PA and dietary live microbes has rarely been studied together. It is hypothesized that a combined approach, integrating both increased PA and dietary intake of live microbes, could have a more substantial impact on childhood obesity than either factor independently.

Therefore, the study aimed to explore the associations between dietary live microbe intake, PA, and the prevalence of overweight or obesity in children, specifically focusing on their combined effects. By addressing this gap, we hope to identify new strategies for promoting healthy weight in children, with the potential to reduce the long-term health risks associated with childhood obesity.

## Methods

### Study design and participants

The NHANES provides a nationally representative sample of the non-institutionalized United States population through a complex, multi-stage probability design. Data for children and adolescents aged 2–17 in this cross-sectional study were sourced from the NHANES database. The study protocol was approved by the Ethics Review Board of the National Center for Health Statistics. Given the de-identified and publicly available nature of the data, the ethical approval statement and the requirement for informed consent were waived for this study.

**Figure 1:**
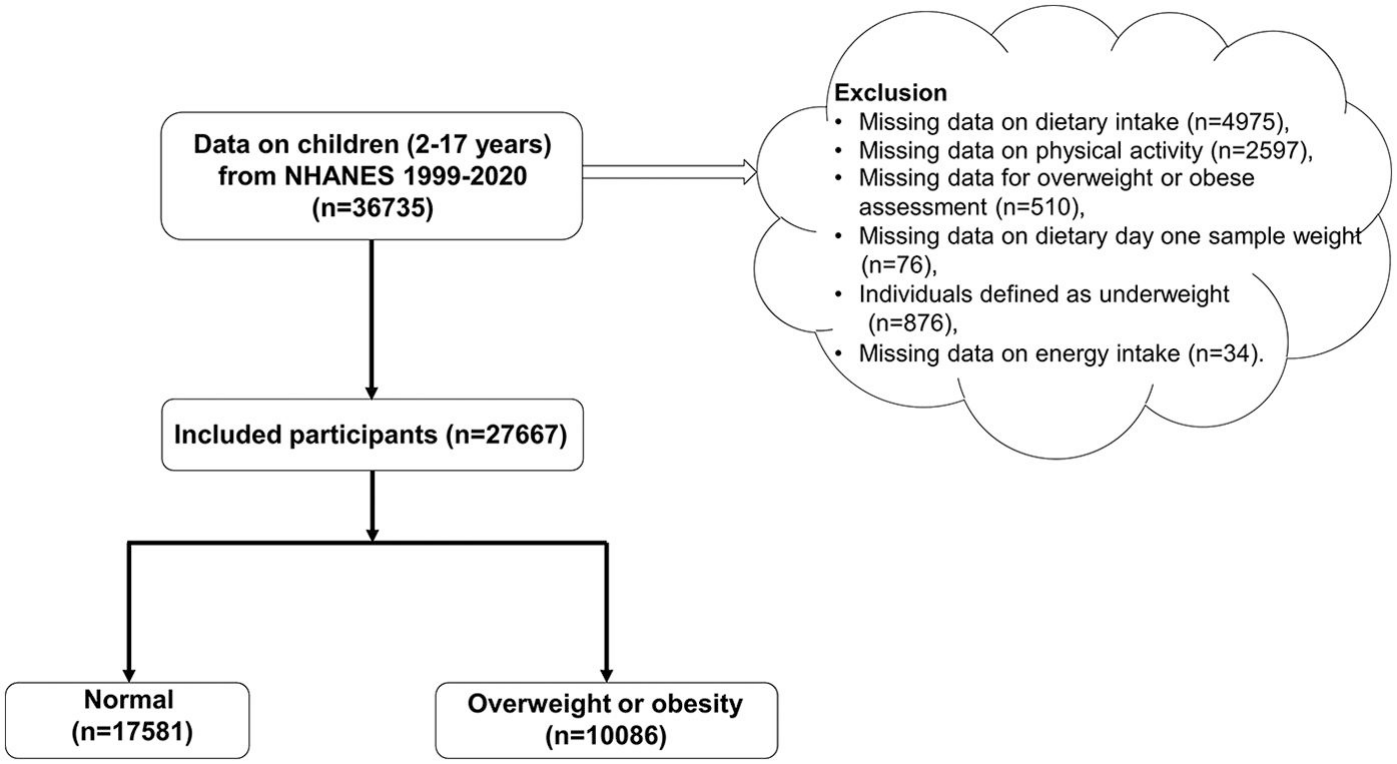
Flowchart of participant selection from the national health and nutrition examination survey, 1999–2020.

Adolescents meeting these criteria were included: (1) aged 2–17 years, (2) assessed for overweight or obesity, (3) complete data on dietary intake and PA. Exclusion criteria included missing data on dietary intake, PA, overweight/obesity status, dietary day one sample weight, or energy intake, and those defined as underweight. The detailed selection process is depicted in Figure 1.

### Dietary live microbe intake

The National Center for Health Statistics linked the 24-h eating data to the United States Department of Agriculture Food Surveys Nutrient Database to estimate energy and nutrient intake. The dietary live microbe intake was estimated based on the classification system introduced by Marco et al. (8). Briefly, a panel of four food science and microbiology experts independently reviewed scientific literature and food processing data to assign preliminary live microbe levels (low: <10^4^ CFU/g, medium: 10^4^–10^7^ CFU/g, high: >10^7^ CFU/g) to NHANES food codes. Experts worked in teams to review food codes, with initial disagreements (occurring for approximately 6%–7% of codes) being resolved through consensus discussions, re-evaluation of evidence, and, when necessary, consultation with an external authority. The underlying assumption for this categorization was that these thresholds represent gradients of microbial exposure, with low-level foods typically being sterile or highly processed, medium-level including many fresh produces and some fermented items, and high-level consisting predominantly of fermented foods (8). Based on this expert-driven system, foods were categorized into the three levels. Additionally, for the purpose of this analysis, the consumption of medium- and high- level foods were combined and classified as “MedHi” intake (15). Using the median consumption value of 251.12 g, live microbe intake was further divided into high and low groups for analysis.

### Physical activity

For children aged 3–11 years, the PA status was assessed using NHANES questionnaires. A score of at least 7 on questions “How many times per week does the child play or exercise enough to make him/her sweat and breathe hard” and “Ding the past 7 days, on how many days was the child physically active for a total of at least 60 min per day? Add up all the time the child spent in any kind of PA that increased his/her heart rate and made him/her breathe hard some of the time”, indicated ideal PA (16, 17). For those aged 12–17 years, PA was measured in metabolic equivalent (MET) minutes of moderate to vigorous PA per week (18). NHANES provides MET values for various exercises, with PA calculated using the formula: PA (MET-min) = MET ×duration of each PA (19). Ideal PA was defined as ≥180 MET-min/day. It should be noted that the assessment of physical activity relied on self-reported questionnaires, which may be subject to recall and social desirability bias.

### Overweight and obesity

The body mass index (BMI)-for-age percentile growth charts are the most commonly used indicator to measure the size and growth patterns of adolescents in the United States (20). Overweight was defined as BMI at or above the 85th and below the 95th sex-specific percentile of the BMI-for-age growth chart from the Centers for Disease Control and Prevention (CDC) (20, 21). Obesity was defined as BMI at or above the sex-specific 95th percentile of the CDC BMI-for-age growth charts (22).

### Covariates

Covariates adjusted in this study include age, sex, race, poverty income ratio (PIR), sedentary time, higher parental education, birth weight, maternal smoking during pregnancy, tobacco exposure, and Vitamin D intake level. The selection of these covariates was based on their known or potential associations with childhood obesity. Age was treated as a continuous variable. Sex and Race/Ethnicity were self-reported and categorized as Mexican American, Non-Hispanic White, Non-Hispanic Black, and Other races, as defined in the NHANES demographic data. Poverty income ratio (PIR) is a ratio of family income to the federal poverty threshold, provided by NHANES. It was categorized as <1 (below poverty level) or ≥1 (at or above poverty level) (23). Sedentary time was determined based on self-reported responses to the questions about time spent sitting while watching TV or videos, using a computer, and other daily sedentary behaviors (24). It was categorized as <4 h/day or ≥4 h/day (24). Higher parental education was defined as the highest education level in the household being above high school level. Birth weight obtained from the questionnaire data and categorized as low (<5.5 lbs), normal (5.5–9 lbs), or high (≥9 lbs). Maternal smoking during pregnancy was a binary variable (yes//no) based on self-report. Tobacco exposure was assessed using serum cotinine levels, a biomarker for nicotine exposure. Participants were classified as exposed (≥0.05 ng/ml) or unexposed (<0.05 ng/ml) (25). Serum vitamin D level was measured in serum and categorized as sufficient (≥20 ng/ml) or insufficient (<20 ng/ml) based on established clinical cut-offs.

### Statistical analysis

In this study, we considered masked variance and used the weighting methodology in all analyses. Continuous variables were expressed as means and standard errors, and the comparison between the normal and overweight or obesity groups was performed with *t*-tests. Categorical variables were described as constitution ratio [*n* (%)], and Chi-square tests were conducted to compare the differences between the two groups. Weighted univariate logistic regression models were performed to select potential covariates. The detailed screening results are illustrated in Supplementary Table 1. The associations between dietary live microbe intake, PA, and overweight or obesity were explored using the weighted multivariate logistic regression models. In addition, we investigated the combined effect of dietary live microbe and PA on overweight or obesity. Odds ratios (ORs) and 95% confidence intervals (CIs) were expressed for the results. The combined effect was also explored in age, sex, and tobacco exposure subgroups. The expression levels of enterodiol, enterolactone, and C-reactive protein (CRP) were also detected in children with different live microbe dietary and PA statuses, using the Kruskal–Wallis rank sum test and Wilcoxon rank sum test. All statistical analyses were conducted utilizing R version 4.3.3, and a two-sided *P*-value <0.05 was considered statistically significant.

## Results

### Characteristics of children aged 2–17 years

In total, 27, 667 children were included in our study. Among them, 10, 086 (36.45%) were overweight or obesity. With a mean age of 9.29 ± 0.05 years, most children were males (50.71%) and non-Hispanic White (57.48%). Only 2, 227 (9.58%) children were in the high dietary live microbe intake group. 20,296 (71.91%) children have ideal PA. Statistical significances were observed in age, sex, race, PIR, sedentary time, higher parental education, birth weight, maternal smoking during pregnancy, tobacco exposure, vitamin D level, type of dietary live microbes, and PA between the two groups (all *P* < 0.05). The baseline characteristics of the study participants, stratified by weight status (normal weight vs. overweight/obesity), are summarized in Table 1.

**Table 1:** Characteristics of included children aged 2–17-year-old.

Variables	Total (*n* = 27,667)	Normal (*n* = 17,581)	Overweight or obesity (*n* = 10,086)	*P*
Age, years, Mea*n* ± S.E	9.29 ± 0.05	8.92 ± 0.06	9.97 ± 0.07	<0.001^a^
Sex, *n* (%)				0.013^b^
Male	14,019 (50.71)	8,915 (49.86)	5,104 (52.29)	
Female	13,648 (49.29)	8,666 (50.14)	4,982 (47.71)	
Race, *n* (%)				<0.001^b^
Mexican American	7,372 (13.75)	4,280 (11.85)	3,092 (17.32)	
Non-Hispanic White	8,011 (57.48)	5,500 (60.10)	2,511 (52.53)	
Non-Hispanic Black	7,569 (14.11)	4,700 (13.43)	2,869 (15.37)	
Other Race	4,715 (14.67)	3,101 (14.61)	1,614 (14.78)	
PIR, *n* (%)				<0.001^b^
<1	8,375 (22.05)	5,164 (20.67)	3,211 (24.65)	
≥1	17,321 (71.94)	11,200 (73.64)	6,121 (68.73)	
Unknown	1,971 (6.01)	1,217 (5.68)	754 (6.62)	
Sedentary time, h, *n* (%)				<0.001^b^
<4	10,314 (39.00)	6,797 (40.89)	3,517 (35.45)	
≥4	15,011 (50.28)	9,304 (48.84)	5,707 (53.00)	
Unknown	2,342 (10.71)	1,480 (10.27)	862 (11.56)	
Energy intake, kcal/day, Mean ± S.E	1,931.75 ± 8.56	1,921.50 ± 9.64	1,951.05 ± 13.56	0.052^a^
Higher parental education, *n* (%)				<0.001^b^
No	13,392 (38.12)	8,114 (36.01)	5,278 (42.09)	
Yes	11,229 (48.80)	7,536 (51.18)	3,693 (44.32)	
Unknown	3,046 (13.08)	1,931 (12.81)	1,115 (13.58)	
Birth weight, lbs, *n* (%)				<0.001^b^
5.5–9	18,678 (68.80)	11,927 (69.42)	6,751 (67.64)	
<5.5	3,053 (10.14)	2,136 (10.85)	917 (8.79)	
≥9	1,945 (7.56)	1,040 (6.54)	905 (9.49)	
Unknown	3,991 (13.50)	2,478 (13.19)	1,513 (14.07)	
Maternal smoking during pregnancy, *n* (%)				<0.001^b^
No	20,855 (74.16)	13,370 (75.24)	7,485 (72.12)	
Yes	3,264 (13.51)	2,014 (12.57)	1,250 (15.28)	
Unknown	3,548 (12.33)	2,197 (12.19)	1,351 (12.60)	
Tobacco exposure, ng/ml, *n* (%)				<0.001^b^
<0.05	10,283 (39.14)	6,353 (38.57)	3,930 (40.22)	
≥0.05	10,637 (36.86)	6,295 (34.57)	4,342 (41.19)	
Unknown	6,747 (24.00)	4,933 (26.86)	1,814 (18.60)	
VID level, ng/ml, *n* (%)				<0.001^b^
<20	5,187 (12.25)	2,688 (9.86)	2,499 (16.75)	
≥20	12,747 (51.63)	8,376 (52.28)	4,371 (50.41)	
Unknown	9,733 (36.12)	6,517 (37.86)	3,216 (32.84)	
Type of dietary live microbes, *n* (%)				0.015^b^
Low	11,925 (40.43)	7,438 (39.46)	4,487 (42.24)	
Median	10,891 (39.24)	6,933 (39.57)	3,958 (38.63)	
High	4,851 (20.33)	3,210 (20.97)	1,641 (19.13)	
Live microbe intake group, *n* (%)				0.004^b^
Low	25,440 (90.42)	16,051 (89.78)	9,389 (91.64)	
High	2,227 (9.58)	1,530 (10.22)	697 (8.36)	
PA, *n* (%)				<0.001^b^
not ideal physical activity	7,371 (28.09)	4,508 (26.73)	2,863 (30.65)	
Ideal physical activity	20,296 (71.91)	13,073 (73.27)	7,223 (69.35)	

S.E, standard error; PIR, poverty income ratio; VID, Vitamin D; PA, physical activity.

a Weighted *t*-test.

b Rao-Scott Chi-square test.

### Associations of dietary live microbe intake, PA, with overweight or obesity in children

To examine the associations of dietary live microbes and physical activity with overweight/obesity, we employed multivariable logistic regression models with sequential adjustments for potential confounders. In model 2, socio-demographical covariates were adjusted including age, sex, and race. High live microbe intake group (OR = 0.82, 95%CI: 0.71–0.95) and ideal PA (OR = 0.74, 95%CI: 0.67–0.82) were associated with decreased odds of overweight or obesity in children. In model 3, all potential covariates were adjusted including age, sex, race, PIR, sedentary time, higher parental education, birth weight, maternal smoking during pregnancy, tobacco exposure, and vitamin D level. High live microbe intake group (OR = 0.83, 95%CI: 0.72–0.97) and ideal PA (OR = 0.76, 95%CI: 0.69–0.84) remain related to a lower incidence of overweight or obesity in children (Table 2).

**Table 2:** Associations between dietary live microbe intake, physical activity, and overweight or obesity in children.

Variables	Model1		Model2		Model3	
	OR (95% CI)	*P*	OR (95% CI)	*P*	OR (95% CI)	*P*
Type of dietary live microbes						
Low	Ref		Ref		Ref	
Median	0.91 (0.83–0.99)	0.049	0.94 (0.86–1.04)	0.221	0.96 (0.88–1.06)	0.425
High	0.85 (0.76–0.95)	0.006	0.93 (0.83–1.04)	0.199	0.96 (0.86–1.08)	0.530
Live microbe intake group						
Low	Ref		Ref		Ref	
High	0.80 (0.69–0.93)	0.004	0.82 (0.71–0.95)	0.010	0.83 (0.72–0.97)	0.020
PA						
not ideal physical activity	Ref		Ref		Ref	
Ideal physical activity	0.83 (0.75–0.91)	<0.001	0.74 (0.67–0.82)	<0.001	0.76 (0.69–0.84)	<0.001

OR, odds ratio; CI, confidence intervals; Ref, reference.

Model 1 was crude model.

Model 2 adjusting age, sex, and race.

Model 3 adjusting age, sex, race, PIR, sedentary time, higher parental education, birth weight, maternal smoking during pregnancy, tobacco exposure, and VID level.

**Figure 2:**
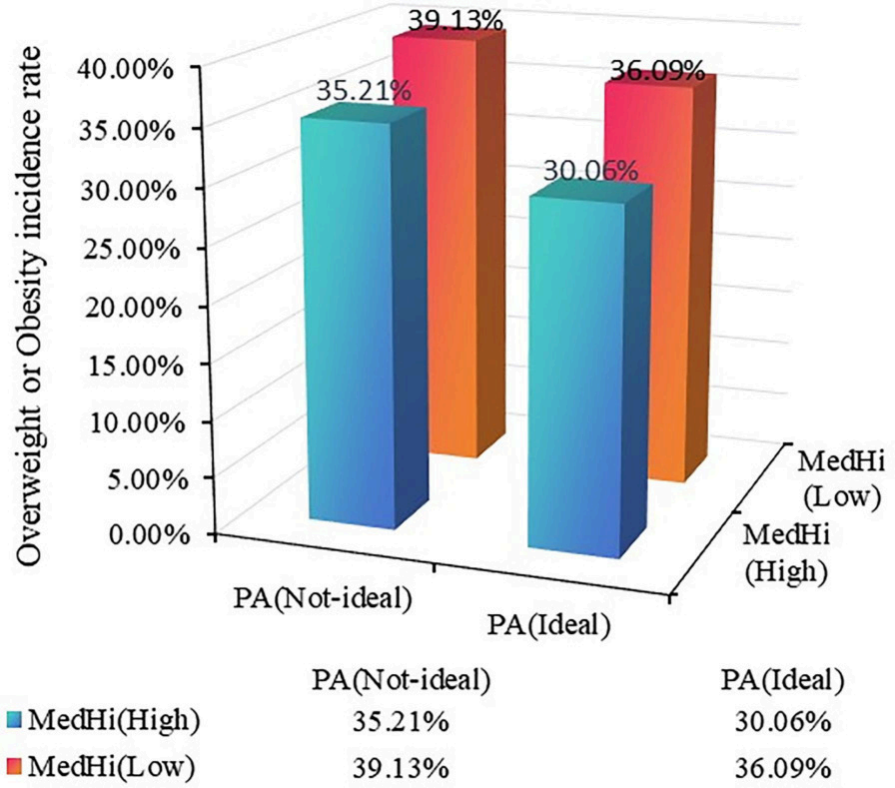
The prevalence of overweight or obesity in different live microbe intake and physical activity groups.

We next investigated the potential synergistic effect of dietary live microbe intake and physical activity by creating a combined exposure variable and then examining this association across key demographic and exposure subgroups. As illustrated in Figure 2, we compare the prevalence of overweight or obesity in children with different live microbe intake levels and PA. Among the four groups, children with high live microbe intake and ideal PA have the lowest prevalence. The children with low live microbe intake and without ideal PA have the highest prevalence of overweight or obesity (*χ*^2^ = 52.311, *P* < 0.001). The results indicated the potential combined effect between dietary live microbe intake and PA. Further analysis found that children with high live microbe intake and ideal PA were associated with decreased odds of overweight or obesity (OR = 0.65, 95%CI: 0.54–0.77, *P* for trend <0.001) when compared with those with low live microbe intake and non-ideal PA (Table 3).

**Table 3:** The combined effect of dietary live microbe intake and physical activity on overweight or obesity in children.

Variables	Model1		Model2		Model3	
	OR (95% CI)	*P*	OR (95% CI)	*P*	OR (95% CI)	*P*
Live microbe intake & PA						
Low-live microbe intake & not ideal physical activity	Ref		Ref		Ref	
High- live microbe intake & not ideal physical activity	0.81 (0.59–1.10)	0.175	0.83 (0.61–1.13)	0.241	0.83 (0.61–1.13)	0.235
Low- live microbe intake & ideal physical activity	0.83 (0.75–0.91)	<0.001	0.74 (0.67–0.82)	<0.001	0.76 (0.69–0.85)	<0.001
High- live microbe intake & ideal physical activity	0.66 (0.56–0.79)	<0.001	0.61 (0.52–0.73)	<0.001	0.65 (0.54–0.77)	<0.001
*P* for trend	<0.001		<0.001		<0.001	

OR: odds ratio; CI: confidence intervals; Ref: reference.

Model 1 was crude model.

Model 2 adjusting age, sex, and race.

Model 3 adjusting age, sex, race, PIR, sedentary time, higher parental education, birth weight, maternal smoking during pregnancy, tobacco exposure, and VID level.

### The combined effect of dietary live microbe intake and PA in diverse age, sex, and tobacco exposure subgroups

To assess the consistency of this combined effect, we conducted stratified analyses by age, sex, and tobacco exposure status. We observed the combined effect of high live microbe intake and ideal PA were also linked to lower incidence of overweight or obesity across different age, sex, and tobacco exposure subgroups (all *P* < 0.05) (Table 4). The findings suggest that high live microbe intake and ideal PA could be universally beneficial in promoting healthy weight, regardless of other influences like age, sex, or tobacco exposure.

**Table 4:** The combined effect of dietary live microbe intake and physical activity in diverse age, sex, and tobacco exposure subgroups.

Group	Variables	Model 1		Model 2		Model 3	
		OR (95% CI)	*P*	OR (95% CI)	*P*	OR (95% CI)	*P*
Age							
2–11	Low- live microbe intake & not ideal physical activity	Ref		Ref		Ref	
	High- live microbe intake & not ideal physical activity	0.81 (0.58–1.14)	0.226	0.84 (0.60–1.16)	0.287	0.85 (0.61–1.19)	0.340
	Low- live microbe intake & ideal physical activity	0.74 (0.66–0.83)	<0.001	0.74 (0.66–0.83)	<0.001	0.75 (0.67–0.84)	<0.001
	High- live microbe intake & ideal physical activity	0.62 (0.50–0.77)	<0.001	0.63 (0.51–0.79)	<0.001	0.66 (0.52–0.82)	<0.001
*P* for trend		<0.001		<0.001		<0.001	
12–17	Low- live microbe intake & not ideal physical activity	Ref		Ref		Ref	
	High- live microbe intake & not ideal physical activity	0.81 (0.34–1.94)	0.633	0.79 (0.34–1.86)	0.595	0.73 (0.31–1.68)	0.449
	Low- live microbe intake & ideal physical activity	0.80 (0.63–1.01)	0.056	0.80 (0.63–1.02)	0.068	0.86 (0.67–1.10)	0.238
	High- live microbe intake & ideal physical activity	0.61 (0.45–0.82)	0.001	0.63 (0.47–0.85)	0.003	0.71 (0.52–0.97)	0.033
*P* for trend		0.001		0.003		0.041	
Sex							
Male	Low- live microbe intake & not ideal physical activity	Ref		Ref		Ref	
	High- live microbe intake & not ideal physical activity	0.89 (0.58–1.37)	0.588	0.90 (0.58–1.37)	0.614	0.91 (0.58–1.41)	0.660
	Low- live microbe intake & ideal physical activity	0.78 (0.68–0.90)	0.001	0.69 (0.60–0.80)	<0.001	0.72 (0.63–0.84)	<0.001
	High- live microbe intake & ideal physical activity	0.59 (0.46–0.75)	<0.001	0.53 (0.41–0.68)	<0.001	0.58 (0.45–0.75)	<0.001
*P* for trend		<0.001		<0.001		<0.001	
Female	Low- live microbe intake & not ideal physical activity	Ref		Ref		Ref	
	High- live microbe intake & not ideal physical activity	0.73 (0.48–1.11)	0.144	0.78 (0.52–1.18)	0.236	0.77 (0.51–1.18)	0.234
	Low- live microbe intake & ideal physical activity	0.85 (0.74–0.98)	0.022	0.79 (0.69–0.90)	0.001	0.80 (0.70–0.92)	0.002
	High- live microbe intake & ideal physical activity	0.74 (0.57–0.96)	0.024	0.70 (0.54–0.92)	0.011	0.73 (0.55–0.95)	0.021
*P* for trend		0.007		<0.001		0.001	
Tobacco exposure							
<0.05	Low- live microbe intake & not ideal physical activity	Ref		Ref		Ref	
	High- live microbe intake & not ideal physical activity	0.84 (0.56–1.24)	0.376	0.87 (0.60–1.28)	0.482	0.82 (0.55–1.22)	0.321
	Low- live microbe intake & ideal physical activity	0.78 (0.67–0.92)	0.002	0.73 (0.61–0.86)	<0.001	0.75 (0.63–0.90)	0.002
	High- live microbe intake & ideal physical activity	0.61 (0.47–0.79)	<0.001	0.58 (0.44–0.76)	<0.001	0.60 (0.45–0.80)	0.001
*P* for trend		<0.001		<0.001		<0.001	
≥0.05	Low- live microbe intake & not ideal physical activity	Ref		Ref		Ref	
	High- live microbe intake & not ideal physical activity	0.90 (0.47–1.70)	0.739	0.90 (0.47–1.71)	0.738	0.89 (0.47–1.71)	0.728
	Low- live microbe intake & ideal physical activity	0.83 (0.72–0.96)	0.013	0.77 (0.66–0.89)	0.001	0.78 (0.67–0.91)	0.002
	High- live microbe intake & ideal physical activity	0.81 (0.57–1.14)	0.223	0.77 (0.55–1.10)	0.146	0.80 (0.56–1.14)	0.216
*P* for trend		0.015		0.001		0.004	

OR, odds ratio; CI, confidence intervals; Ref, reference.

Model 1 was crude model.

Model 2 adjusting age, sex, and race.

Model 3 adjusting age, sex, race, PIR, sedentary time, higher parental education, birth weight, maternal smoking during pregnancy, tobacco exposure, and VID level.

For subgroup analysis, the corresponding covariates were not adjusted in its subgroups.

### The expression of enterodiol, enterolactone, with CRP in children with different combined groups of dietary live microbe intake and PA

**Figure 3:**
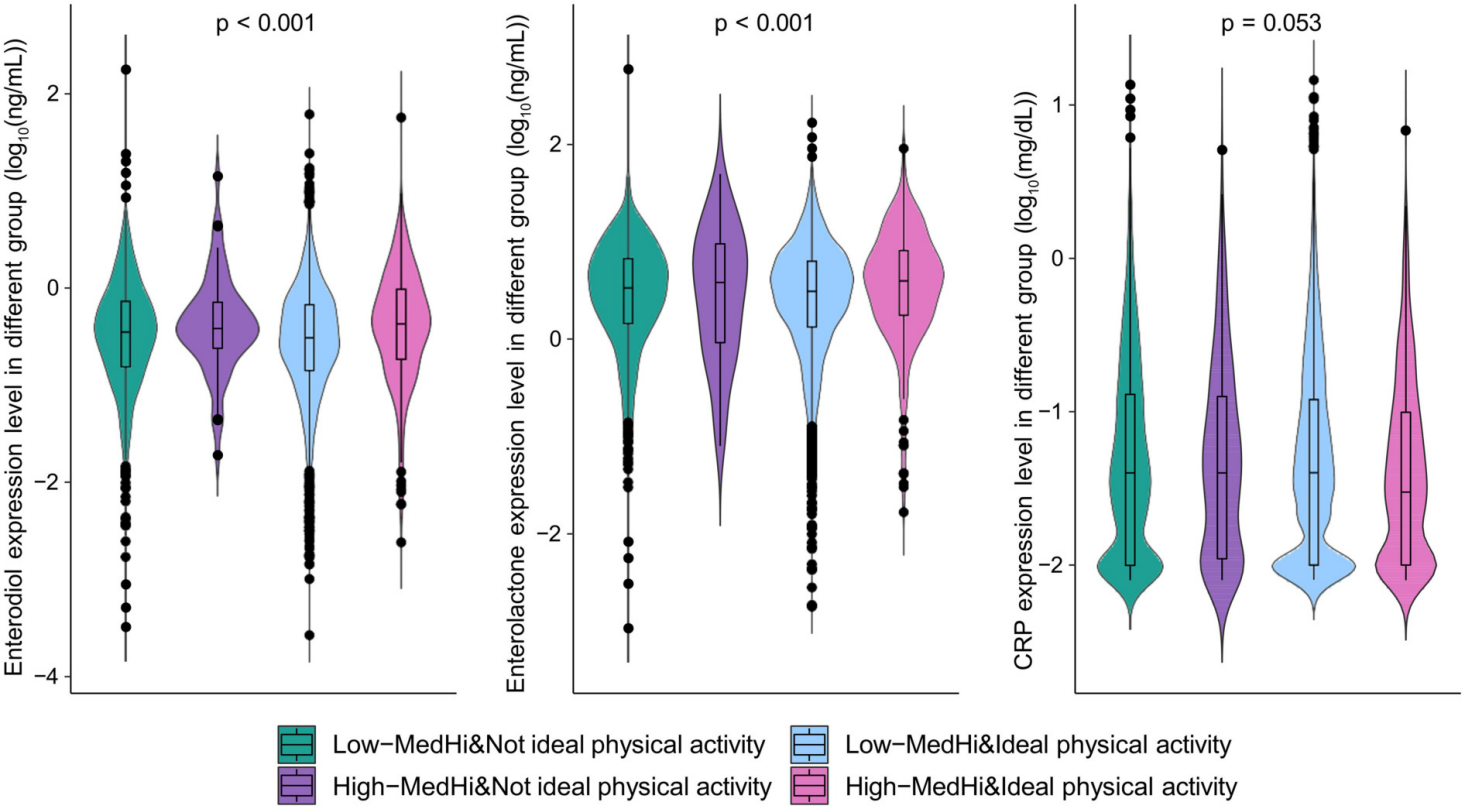
The expression of enterodiol, enterolactone, and CRP in different live microbe intake and physical activity groups.

To explore potential biological mechanisms underlying the observed associations, we compared the levels of gut microbiota-derived metabolites (enterodiol, enterolactone) and a systemic inflammation marker (CRP) across the combined dietary and physical activity groups (Figure 3). The expression levels of enterodiol and enterolactone were the highest in the high live microbe intake and ideal PA group, indicating abundant intestinal microbial in this group. There was no statistical significance on the CRP expression among the four groups. The comparison between each group found that CRP expression levels were lowest in high live microbe intake and ideal PA group (compared to low live microbe intake and non-ideal PA group, *P* = 0.008; compared to high live microbe intake and non-ideal PA group, *P* = 0.054; compared to low live microbe intake and ideal PA group, *P* = 0.018).

## Discussion

Our findings suggested that a high intake of live microbe and ideal PA levels were associated with lower odds of being overweight or obese. Furthermore, children who had both high live microbe intake and engaged in ideal PA exhibited the lowest prevalence of overweight or obesity. Importantly, the findings suggested a combined effect between live microbe intake and PA on body weight. In addition, children in the high live microbe and ideal PA group had the highest levels of enterodiol and enterolactone, which are intestinal microbial markers, and the lowest levels of CRP, an inflammatory marker. The findings highlight the potential synergistic role of diet and physical activity in preventing childhood overweight and obesity.

Our findings regarding the independent beneficial effects of dietary live microbes and PA align with a substantial body of literature (10, 26, 27). For instance, a randomized controlled clinical trial demonstrated that specific probiotic strains (HY7601 and KY1032) exert anti-obesity effects by modulating the gut microbiota (28), which resonates with our observation of a protective association. Similarly, the well-established role of PA in improving insulin sensitivity and reducing adiposity (29–31). However, the novel and central findings of present study is the significant combined effect between these factors. Directly comparable studies on this specific interaction in children are scarce. Our analysis suggests that the combined effect of high live microbes intake and ideal PA was associated with 35% lower odds of overweight/obesity (OR = 0.65). This suggests that studying these lifestyle factors in isolation, as most prior research has done, may underestimate their true public health potential. There are several reasons why previous studies may not have captured this synergistic effect. First, many interventions or observational studies focus exclusively on either diet or physical activity, lacking the statistical power or design to test for interactions. Second, the quantification of “dietary live microbes” is a relatively recent advancement (8). Without this standardized classification, previous studies could not precisely examine its role and interaction with other factors. Therefore, our study moves beyond the established independent effects and provides among the first pieces of large-scale, cross-sectional evidence supporting a combined lifestyle approach targeting both the gut microbiome and physical activity for pediatric obesity prevention.

Our findings of a combined effect invite an exploration of the potential bidirectional mechanisms linking dietary live microbes, PA, and the gut microbiota. We propose a physiological mechanisms framework wherein these factors interact with each other to combat obesity. On one hand, PA may create a more favorable gut environment for dietary live microbes to exert their benefits (32, 33). Furthermore, PA has shown to increase microbial diversity and the abundance of specific bacteria that produce short-chain fatty acids life butyrate (34). Butyrate not only reduces systemic inflammation and improves insulin sensitivity but also serves as the primary energy source for colonocytes, reinforcing gut barrier integrity (35). A stronger gut barrier prevents the translocation of pro-inflammatory bacterial fragments into the bloodstream, a key driver of obesity-related metabolic inflammation. Thus, PA may “prepare the ground” for ingested live microbes by fostering a healthier and more receptive gut ecosystem. On the other hand, a live microbe-rich diet may augment the metabolic benefits of PA. The short-chain fatty acids produced by gut microbiota from dietary fiber and other substrates can influence host energy metabolism. For instance, they can stimulate the release of gut hormones such glucagon-like peptide-1 (GLP-1), which promotes satiety and improves glucose homeostasis-effects that complement the improvements in insulin sensitivity induced by PA (36). Some microbial metabolites are also involved in mitochondrial biogenesis and fatty acid oxidation in skeletal muscle, potentially enhancing exercise capacity and recovery. Therefore, the synergy we observed may stem from a virtuous cycle: PA enriches a gut environment conducive to the beneficial actions of dietary microbes, while these microbes and their metabolites, in turn, amplify the metabolic and anti-inflammatory rewards of PA. This integrated model provides a compelling explanatory framework for our results and highlights the necessity of combining both lifestyle interventions for maximal effect in pediatric obesity prevention.

Enterodiol and enterolactone are not merely markers of microbial presence; they are bioactive metabolites produced specifically by the gut microbiota from dietary lignans found in whole grains, fruits, and vegetables (37). Their elevated levels in our combined group serve as an indicator of a functionally active and health-promoting gut microbiota. Byeong their known anti-inflammatory and antioxidant properties, these enterolignans have been specifically implicated in body weight regulation. Mechanistically, they are thought to modulate fat metabolism by activating peroxisome proliferator-activated receptors, which promote fatty acid β-oxidation and inhibit adipocyte differentiation, thereby reducing fat storage (38, 39). Conversely, CRP is a key mediator and marker of the low-grade systemic inflammation that is a hallmark of obesity pathophysiology. Adipose tissue, especially in obesity, secretes pro-inflammatory cytokines, which in turn stimulate hepatic production of CRP. Elevated CRP perpetuates a vicious cycle by further promoting insulin resistance and disrupting normal lipid metabolism, facilitating further weight gain and metabolic dysfunction (40). The significant reduction in CRP levels in the combined group indicates a potent attenuation of this obesity-driving inflammatory cascade. This anti-inflammatory effect likely stems from the combined actions of exercise-induced myokines and microbe-derived anti-inflammatory metabolites.

Given the increasing prevalence of overweight and obesity in children worldwide, identifying modifiable risk factors such as diet and PA is crucial for developing effective prevention strategies. Our study suggests that encouraging children to consume a diet rich in live microbes (e.g., through probiotics or fermented foods) and engage in regular PA may offer a complementary approach to obesity prevention. The observed reduction in CRP levels and the increase in intestinal microbial markers, such as enterodiol and enterolactone, provide a plausible biological basis for this synergy. To translate these findings into actionable strategies, policymakers and public health stakeholders could consider: (1) integrating the promotion of live-microbe-rich foods and regular physical activity into public health guidelines and educational campaigns; (2) fostering school-based programs that improve access to these foods and ensure adequate opportunities for physical activity; (3) prioritizing further research to strengthen the evidence base for these combined interventions. Incorporating these integrated lifestyle factors into public health initiatives and pediatric care guidelines could represent a promising strategy to help address the obesity epidemic.

Nevertheless, there are several limitations in our study that warrant consideration. First, the cross-sectional nature of our study precludes the determination of causality. The associations observed between dietary live microbe intake, physical activity, and overweight/obesity may be subject to reverse causation; for instance, children with obesity might alter their dietary habits or physical activity levels. Therefore, our results should be interpreted as identifying important associations rather than establishing causal links. Second, although we adjusted for a wide range of potential confounders, the possibility or residual confounding cannot be ruled out. Unmeasured factors, such as genetic predisposition, detailed dietary patterns beyond live microbes (e.g., total sugar or fiber intake), and psychosocial factors, might influence both the exposures and the outcome. This could mean that the protective effects we observed might be over- or under-estimated. Third, the assessment of physical activity relied on self-reported questionnaires, which are susceptible to recall and social desirability bias. This measurement bias could lead to the misclassification of participants’ true activity levels, which likely attenuates the observed associations toward the null, meaning the true protective effect of physical activity might be stronger than what we reported. Finally, regarding the generalizability of our findings, the NHANES data are nationally representative of the non-institutionalized US population. Therefore, our results are likely generalizable to children and adolescents in the United States with similar demographic characteristics. However, caution should be exercised when extrapolating these findings to populations with substantially different genetic backgrounds, dietary cultures, or built environments that influence physical activity. The specific food sources and definitions of “dietary live microbes” are based on the US food supply, which may not directly translate to other countries.

Beyond the methodological considerations, translating these findings into real-world public health recommendations faces several practical challenges. First, to some extent, ensuring consistent adherence to a diet rich in live microbes can be difficult. Factors such as taste preferences, dietary habits established in childhood, and the higher relative cost and lower accessibility of certain fresh and fermented foods in underserved communities are significant barriers. Second, promoting and maintain ideal levels of physical activity requires addressing issues related to the increasing sedentary lifestyle, screen time, and access to safe recreational spaces. Therefore, successful implementation would likely require multi-level strategies that extend byyond individual education. These could include public health policies to improve the affordability and availability of healthy foods, creating more community-based physical activity programs, and educational initiatives that engage both children and their families to foster sustainable lifestyle changes.

## Conclusion

Our study provides evidence that the combination of adequate dietary live microbe intake and ideal physical activity offers stronger protection against overweight and obesity in children and adolescents than either factor alone. This finding fills an important gap in the pediatric literature by highlighting a synergistic approach to obesity prevention. To translate these findings into practice, future public health strategies and clinical advice should move beyond isolated recommendations and promote the integrated adoption of both behaviors. While further longitudinal studies are warranted to establish causality and elucidate the underlying mechanisms, our findings underscore the potential of a combined dietary and physical activity strategy as a foundational element in combating childhood obesity.

## Data Availability

Publicly available datasets were analyzed in this study. This data can be found here: NHANES, https://wwwn.cdc.gov/nchs/nhanes/.
