# Supplementary material for: The combined effect of dietary live microbe intake and physical activity on overweight or obesity in children and adolescents aged 2–17 years: a cross-sectional study from the NHANES
Source: Front Pediatr. 2025 Dec 1;13:1653786. doi: 10.3389/fped.2025.1653786 (PMC12702851; doi:10.3389/fped.2025.1653786)
Supplement: Supplementary file 1 [file Table1.docx]

Table S1 Selection of the potential covariates

| Variables | Outcome/Total | OR (95% CI) | *P* |
| --- | --- | --- | --- |
| Age | 10086/27667 | 1.05 (1.04-1.06) | <0.001 |
| Sex |  |  |  |
| Male | 5104/14019 | Ref |  |
| Female | 4982/13648 | 0.91 (0.84-0.98) | 0.013 |
| Race |  |  |  |
| Mexican American | 3092/7372 | Ref |  |
| Non-Hispanic White | 2511/8011 | 0.60 (0.55-0.66) | <0.001 |
| Non-Hispanic Black | 2869/7569 | 0.78 (0.71-0.87) | <0.001 |
| Other Race | 1614/4715 | 0.69 (0.62-0.77) | <0.001 |
| PIR |  |  |  |
| <1 | 3211/8375 | Ref |  |
| ≥1 | 6121/17321 | 0.78 (0.72-0.86) | <0.001 |
| Unknown | 754/1971 | 0.98 (0.81-1.18) | 0.799 |
| Sedentary time |  |  |  |
| <4 | 3517/10314 | Ref |  |
| ≥4 | 5707/15011 | 1.25 (1.15-1.37) | <0.001 |
| Unknown | 862/2342 | 1.30 (1.11-1.52) | 0.001 |
| Energ intake | 10086/27667 | 1.00 (1.00-1.00) | 0.051 |
| Higher parental education |  |  |  |
| No | 5278/13392 | Ref |  |
| Yes | 3693/11229 | 0.74 (0.68-0.81) | <0.001 |
| Unknown | 1115/3046 | 0.91 (0.79-1.04) | 0.157 |
| Birth weight |  |  |  |
| 5.5-9 | 6751/18678 | Ref |  |
| <5.5 | 917/3053 | 0.83 (0.72-0.95) | 0.009 |
| ≥9 | 905/1945 | 1.49 (1.29-1.73) | <0.001 |
| Unknown | 1513/3991 | 1.09 (0.97-1.23) | 0.132 |
| Maternal smoking during pregnancy |  |  |  |
| No | 7485/20855 | Ref |  |
| Yes | 1250/3264 | 1.27 (1.13-1.42) | <0.001 |
| Unknown | 1351/3548 | 1.08 (0.96-1.21) | 0.212 |
| Tobacco exposure |  |  |  |
| <0.05 | 3930/10283 | Ref |  |
| ≥0.05 | 4342/10637 | 1.14 (1.03-1.26) | 0.009 |
| Unknown | 1814/6747 | 0.66 (0.59-0.74) | <0.001 |
| VitaminD level |  |  |  |
| <20 | 2499/5187 | Ref |  |
| ≥20 | 4371/12747 | 0.57 (0.51-0.63) | <0.001 |
| Unknown | 3216/9733 | 0.51 (0.46-0.57) | <0.001 |

OR: odds ratio; CI: confidence intervals; Ref: reference
